# Rho GTPases Signaling in Zebrafish Development and Disease

**DOI:** 10.3390/cells9122634

**Published:** 2020-12-08

**Authors:** Marie-José Boueid, Aya Mikdache, Emilie Lesport, Cindy Degerny, Marcel Tawk

**Affiliations:** Université Paris-Saclay, Hôpital Kremlin Bicêtre, U1195, Inserm, 94276 Le Kremlin Bicêtre, France; marie-jose.boueid@inserm.fr (M.-J.B.); aya.mikdache@curie.fr (A.M.); emilie.lesport@inserm.fr (E.L.)

**Keywords:** zebrafish, small GTPases, Rac, Cdc42, RhoA, GEF, GAP

## Abstract

Cells encounter countless external cues and the specificity of their responses is translated through a myriad of tightly regulated intracellular signals. For this, Rho GTPases play a central role and transduce signals that contribute to fundamental cell dynamic and survival events. Here, we review our knowledge on how zebrafish helped us understand the role of some of these proteins in a multitude of in vivo cellular behaviors. Zebrafish studies offer a unique opportunity to explore the role and more specifically the spatial and temporal dynamic of Rho GTPases activities within a complex environment at a level of details unachievable in any other vertebrate organism.

## 1. Introduction

Rho GTPases, a subgroup of the Ras superfamily, are key molecular switches that transduce signals from the cell surface to major intracellular signaling pathways. Their first members were discovered in 1985 and since then they became the center of intensive studies, showing their implication in a wide range of fundamental cellular processes. This includes cytoskeletal dynamics and re-arrangements, cell migration and polarity, membrane trafficking, NADPH (nicotinamide adenine dinucleotide phosphate) oxidase activation and transcription. Most Rho GTPases alternate between an inactive state when bound to GDP and an active conformation when bound to GTP. The GDP-GTP cycle is mediated by GEFs (guanine nucleotide exchange factors) while GTP hydrolysis is promoted by GAPs (GTPase activating proteins). Once activated, they interact with their downstream effectors to induce diverse cellular responses [1,2].

Most of our knowledge regarding the dynamic activity of Rho GTPases in vertebrates derive mainly from cell culture and in vitro methods, limiting insight into how Rho GTPases function in vivo. Zebrafish or *Danio rerio* has emerged as a very powerful model for in vivo developmental studies. Owing to their transparency and external development, zebrafish embryos are amenable to signaling pathways studies through mRNA or DNA injection, pharmacological treatment and live imaging. Several studies, that we will detail further below, have now shown essential roles for small GTPases in embryonic development and wonderful tools have been applied to zebrafish to unravel further details regarding the temporal and subcellular activity of these Rho GTPases in vivo.

The role of Rho GTPases in development is well described in mammals and it is relatively recently that zebrafish became a chief model to study the mechanisms of Rho GTPases function in vertebrates in vivo. Thirty-two Rho genes have been identified in zebrafish that represent homology to 17 human genes [3], however, overall research has so far focused on three members, Rac1, Cdc42 and RhoA. In this review, we will mainly highlight the many different roles of zebrafish Rac1, Cdc42 and RhoA that have been studied so far. We will also summarize the latest advances in imaging, genetic and pharmacological tools to investigate their function during development and disease in zebrafish.

## 2. Rac1 in Zebrafish Development

As introduced above, Rac1 is one of the most studied small Rho GTPases and a plethora of data has revealed an essential role for zebrafish Rac1 in cell cytoskeletal rearrangement and motility in different cell types including mesodermal cells, endothelial cells and neurons. In this way, Rac1 controls fundamental morphogenetic processes during development that rely on substantial cell movement and cellular reorganization.

### 2.1. Rac1 and Cell Motility in Different Cell Types

During the early development in fish and amphibians, the embryonic body is shaped through gastrulation. This involves extensive cell movements including epiboly, cell internalization and convergence-extension (CE). The first elegant demonstration of a role for Rac1 in the dorsal migration of lateral cells during zebrafish gastrulation was described by Hammerschmidt laboratory [4]. Cell transplantation experiments, that generate chimeric larvae, have shown an autonomous role for Rac1 in promoting lamellipodia formation in these migrating cells downstream of the hyaluronan synthetizing enzyme 2 (Has2). Cellular extensions were visualized using membrane localized GFP. Yu-Long Li et al. have also proposed a strong link between Rac1 signaling and F-actin organization, downstream of PI3K, that coordinates cell movements during epiboly progression [5].

Other studies highlighted a role for Rac1 in the extension of the embryonic dorsal axis and migration of the presomitic mesoderm in zebrafish downstream of p120 Catenin [6]. Further novel and exciting data revealed that the axial mesendoderm follows a true collective process of migration that is mediated by E-cadherin, Wnt-PCP signaling and Rac1 [7]. Drawing on four-dimensional imaging with detailed cell morphology analysis and delicate modification of cellular environment, Dumortier et al. showed a requirement for Rac1, as an intrinsic directionality signal, in collective mesendoderm migration. It has also been shown that Rac1 acts downstream of the TORC2 (Target of Rapamycin Complex 2) component, Sin1, to ensure the migration of the anterior most mesoderm [8].

Endoderm is one of the three germ layers that also needs to internalize and expand over the entire embryo. Live analysis of endodermal cells led by Nicolas David and his colleagues, revealed an active, oriented and actin-based migration that drives these cells to their inner most position. This process was also dependent on Rac1 activity [9]. This followed an original work published by Stainier’s laboratory showing a requirement for Nodal signaling in endodermal cell motility and actin dynamics via Rac1. In this study, the authors generated a *Tg(sox17:GFP-UTRN)* that labels actin-based structures in endodermal cells in order to monitor their behavior. Furthermore, the authors were able to measure Rac1 activity specifically in these cells thanks to a fluorescent Rac1 probe, RFP-PBD, adding to the originality of this work [10].

Studies in mouse embryos showed an important role for Rac1 in mediating intracellular signals required for early gastrulation such as PI3K-Akt and Nap1/WAVE complex [11], and zebrafish embryos revealed precise features of cell dynamics during early morphogenesis, as well as valuable information regarding the spatial activity of Rac1 in vivo with unprecedented level of details.

Neurulation is another complex morphogenetic event that requires extensive cell movement of neural plate cells as well as a direct interaction with underlying mesoderm and surrounding extracellular matrix to coordinate this process [12,13,14]. While the importance of Rac1 is well documented in regulating the fusion of neural folds during mouse neurulation [15], there is no published data so far linking Rac1 to zebrafish neurulation. The only small GTPase to be studied in this context is the R-Ras [16]. *rras* morphants presented bilateral lumens in the spinal cord, however, the same phenotype was not observed in *rras* mutants due to possible redundancy with other *ras* family members.

One of the main features of embryonic development is the correct and coordinated movement of cells in both time and space. Neural crest (NC) cells are one of the most dynamic developmental cells that travel along and explore the embryo with highly organized migratory abilities. Studies in mice have shown an important role for Rac1 in post-migratory NC cells during craniofacial and cardiac development [17], while Helen Matthews and her colleagues unraveled novel and exciting findings regarding the role of small GTPases in NC migration using zebrafish and xenopus embryos. They applied for the first time, in vivo, FRET analysis of these small GTPases. By injecting FRET (fluorescence resonance energy transfer) probes for Rac and RhoA in embryos, they were able to monitor their localization and activity by means of fluorescence, revealing a role for Wnt/PCP and Syndecan 4 in polarizing Rac activity at the front of NC cell to ensure its directional migration [18]. Another exciting study led by Elena Kardash and her colleagues revealed a role for Rac1 in the motility of the highly migratory embryonic germ cells that migrate as individual cells from where they are specified to where they develop in gonads. By applying FRET based strategy similar to the one applied to NC cells, the authors were able to follow Rac activity during germ cells migration in zebrafish embryos while simultaneously monitoring actin dynamics using the *Tg (EGF-actin-nos1-3′UTR).* This elegant study uncovered a crucial role for Rac in controlling the amount and distribution of actin brushes within germ cells that is required for their proper migration [19]. Rac activity was also found to be required downstream of the Gβγ signaling to regulate actin cytoskeleton dynamics in germs cells, similar to its role in gastrulation downstream of Has2 [4,20].

Neutrophils are also highly dynamic cells that migrate towards sites of infections or inflammation where they exert their function. Here again, Rac activity was found to be sufficient to direct the migration of neutrophils in vivo, downstream of PI3K. In this study, the authors generated a photoactivated form of Rac that is spatially and temporally controlled (*Tol2-MPO-mcherry-PA-Rac1-polyA*) and were able to photoactivate Rac in neutrophils in live zebrafish embryos [21].

### 2.2. Rac1 in Cell Rearrangement and Survival

Adding to the original role of Rac1 in cell motility, other studies have unraveled important functions for zebrafish Rac1 in a multitude of developmental processes such as cellular rearrangements within endothelia, axonal and neurite outgrowth, neuronal survival, glial cytoskeletal remodeling and kidney development.

Blood vessel development and growth is a very delicate process that plays a fundamental role not only during embryonic growth but also in many disease conditions such as cancer. Understanding the mechanisms underlying this process is therefore of huge medical importance. Rac1 was found to be required within endothelial cells during vascular development in mice [22], but further exciting details were unraveled thanks to zebrafish. The latter allows a direct and live visualization of angiogenesis and vascular remodeling during development. Indeed, Ilkka Paatero and his colleagues took advantage of zebrafish embryos transparency to uncover a novel role for Rac1 in junction based lamellipodia oscillation and elongation, that helps endothelial cells to move over each other and ensures endothelial rearrangements during angiogenesis [23]. This is consistent with another study led by Daniel Epting and colleagues showing a role for Elmo1 (engulfment and cell motility 1) /Dock180 (ELMO1-dedicator of cytokinesis 1) complex in angiogenesis and lymphangiogenesis upstream of Rac1 [24]. The same group has also uncovered another function for Rac1 in maintaining endothelial cell survival downstream of the Elmo1/Dock180 complex [25]. This highlights a role for Rac1 not only in endothelial cell rearrangements but also in their survival via the PI3K/Akt pathway.

Several studies pointed to a role for Rac1 in mouse neuronal development, such as neural migration, proliferation and survival as well as axonal growth and guidance [26,27,28,29,30]. However, new insight into neuronal Rac1 function was revealed when Yide Zhang and his colleagues applied and optimized super resolution microscopy to zebrafish embryos. Taking advantage of the photoactivable form of Rac, the authors were able to image DRG neurons, using *sox10* driven transgenics, and enhance Rac1 activity specifically within pioneer neurons [31]. Longer filopodia were observed in neurons expressing photoactivated Rac1 with excessive primary neurite formation. The same strategy was followed by the same group to show a role for Rac1 within the growth cones of pioneer axons that controls their spinal cord entry [32]. Along the same lines, a very recent and extremely elegant study using optogenetic axon guidance in zebrafish was particularly exciting. It highlighted a powerful role for Rac1 in repairing defective axonal architecture and function along motor neurons trajectories. The authors generated a new transgenic line expressing a photoactivable form of Rac1 within motor neurons and by stimulating Rac1 using light illumination, axons of the caudal primary motor neurons were then guided across repulsive somitic boundaries. Moreover, by stimulating Rac1, the authors were able to rescue axonal guidance and functional defects observed in pathological environment, for example in *plod3* mutants that show severe axonal growth defects [33]. Further evidence coupling Rac1 activity and axonal regeneration was revealed by Toru Matsukawa and his colleagues using zebrafish retinal explants. The authors propose a link between Wnt signaling activity (Wnt5b, Wnt10a) and the activation of Rac1 during early events of optic nerve regeneration [34].

**Figure 1:**
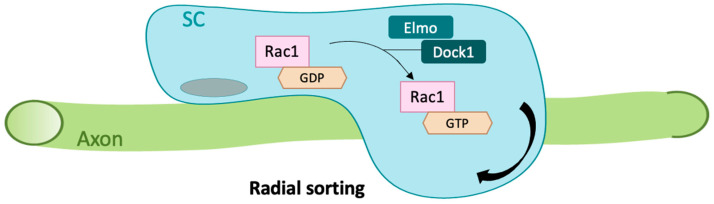
Role of Elmo1/Rac1 signaling during zebrafish Schwann cell development. Elmo1 acts in association with Dock1 to enhance Rac-specific guanine nucleotide exchange factor (GEF) activity of Dock1 by mediating a GDP to GTP exchange on Rac1. Elmo1 function is linked to Rac1 activity within Schwann cells and contributes to the radial sorting process in which a Schwann cell associates with a single axonal segment via cytoskeletal rearrangements. SC, Schwann cell.

Furthermore, and during the neuronal migration process itself, there is a clear requirement for Rac1. Zebrafish Rac1 activity was found to be required for neuronal migration in zebrafish hindbrain rhombic lip cells under the control of the inhibitors of apoptosis proteins (IAPs) [35]. Rac1 also appears to be important for neuronal survival, this is highlighted through its ability to restore neuronal numbers in the trigeminal ganglion in a knockdown zebrafish model of *diras1a* and/or *diras1b,* which belong to a distinct subfamily of Ras GTPases [36]. A recent study by Mikdache et al., showed a fundamental role for the Rac1 regulator Elmo1 in cell survival within the posterior lateral line ganglia (PLLg). The authors propose a strong link between Elmo1 activity and Rac1 since they could restore neuronal numbers either by inhibiting apoptosis or via Rac1 activation in *elmo1* mutants [37]. Furthermore, the authors showed a requirement for Elmo1 not only in neurons but also within peripheral glial cells for early radial sorting, a process that requires extensive cytoskeletal rearrangements and allows Schwann cells to isolate an axon from within a bundle. Here again, Elmo1 function was mediated by Rac1 activation (Figure 1). An indirect link between Pard3 (Par3 family cell polarity regulator) knock-down and a decrease in Rac1 activity has also been described in zebrafish larvae that resulted in abnormal Schwann cell wrapping and a significant reduction in *mbp* expression [38].

At the subcellular level, it has been shown that the Elmo1-Dock1-Rac1 complex is required for ciliogenesis in zebrafish by influencing Ezrin phosphorylation and therefore orchestrating ciliary basal body migration, docking and positioning [39].

### 2.3. Rac1 in Pathology

As detailed above, Rac1 is critical for regulating key cellular processes such as cell migration and survival in different cell types. Clearly, these essential cellular functions mediated by Rac1 could be exploited in pathological conditions such as cancer, whereby cells rely on Rac1 to invade surrounding tissues [40]. One such example is malignant gliomas that account for around 70% of malignant primary brain tumors. IH Jung and his colleagues studied the role of Rac1 in glioma by generating new zebrafish transgenic lines expressing dominant active forms of Akt1 and Rac1 [41]. They showed an important role for active Rac1 in promoting Akt signaling in gliomagenesis. Consistent with this result, a knock-down of Rac1 was sufficient to reduce induced glioblastoma spheroid cells in a zebrafish xenotransplantation model of U373-MG tumorsphere cells [42]. By constitutively expressing an activated form of Rac1 (Rac1V12) along with oncogenic Ras through adulthood, Lucy Dalton and her colleagues showed an essential role for Rac1 in accelerating tumor nodule formation in a zebrafish model of melanocyte neoplasia [43]. Daniela Araiza-Olivera and her colleagues explored potential signaling pathways linked to Rac1 driven malignant melanoma during zebrafish embryonic development. The rasopathy-like phenotypes observed in zebrafish embryos following injection of the human Rac1^P29S^ activating mutation were abolished by PAK and MEK inhibitors in vivo [44]. Indeed, several human Rac1 missense mutations were identified and linked to developmental disorders [45]. Overexpression of the p.Cys18Tyr and p.Asn39Ser Rac1 variants in zebrafish embryos resulted in microcephaly and reduced neural proliferation, therefore functioned as dominant negative mutations [45]. Another interesting finding reported a link between Rac1 activity and glomerulotubular nephropathy, however, in this case, activating Rac1 partially rescued the development of pronephric cysts resulting from *fat1* knockdown, knowing that recessive mutations of the protocadherin *FAT1* cause renal disease in humans [46]. This suggests that this pathogenesis is mediated by a decrease in Rac1 activity, therefore underscoring the importance of a balanced Rac1 signaling in preventing numerous human diseases. Xiaoyang Wan and his colleagues have generated valuable zebrafish tools to assess the role of Rac1 in podocyte injury highlighting a detrimental role for excessive Rac1 activity in podocytes [47]. It was also reported that Rac1 inhibitors exert a protective effect against *arhgdia* (Rho GDP-dissociation inhibitor 1) knockdown in zebrafish larvae, given that ARHGDIA mutation is responsible of nephrotic syndrome, linking the latter to defective Rho GTPase signaling [48].

It is important to note that the only available zebrafish loss of function mutant of the Rac family is the *rac2* mutant, that renders mutant embryos susceptible to infection and can be rescued by ectopic expression of Rac2 or Rac1 specifically in neutrophils, suggesting overlapping function of the two isoforms [49]. Given (i) the effective role of Rac proteins in driving neutrophil migration and activity and (ii) that a dominant negative mutation in Rac2 is associated with human immunodeficiency [50], Alan Hsu and colleagues have tested the hypothesis whether reducing *rac2* activity in zebrafish neutrophils would reduce immunological damage caused by systemic inflammation [51]. For this, the authors generated a new transgenic line that overexpress microRNA-722 in neutrophils and downregulates the transcript level of *rac2.* Indeed, neutrophil-specific miR-722 overexpression protected zebrafish from systemic inflammation and showed an increase resistance to bacterial-induced inflammation highlighting a beneficial outcome for reducing Rac2 levels to dampen neutrophilic inflammation [51].

## 3. Cdc42 in Zebrafish Development

Similar to Rac1, Cdc42 is another Rho GTPase that is well studied and therefore one of the best characterized members of the Rho Family. Mice with a constitutive knockout die at the implantation stage, so conditional deletions were generated to better understand CDC42 function and its signaling pathways [52]. Many studies have been made in human cells or in mouse model [53,54], but less is known in zebrafish development and its normal physiology.

According to phylogenetic analysis and exon organization, three zebrafish sequences were assigned as CDC42 homologs [3]. *cdc42a* (RefSeq: NM_200632) is 99% identical to human *CDC42* and contains nuclear localization and prenylation modification signals. The other two predicted proteins, named Cdc42l2 (Cdc42b) and Cdc42l (Cdc42c), are 77 and 90% identical to human CDC42, respectively. Expression analysis by RT-qPCR showed that *cdc42a* and *c* were expressed during development and in adult zebrafish [3] whereas *cdc42b* was not detected at any timepoint [55].

### 3.1. Cdc42 in Cell Movement and Rearrangement

#### 3.1.1. Gastrulation, Cell Migration

*cdc42* early expression correlates with a role for Cdc42 in gastrulation. Indeed, Cdc42 is already known to control gastrulation in sea urchin embryo [56] and in Xenopus [57], showing a well conserved function across species. As mentioned above, Rac1 activity was shown to be required for gastrulating mouse embryo [11], however less is known of Cdc42 function in this process in vivo. Studies on zebrafish embryos have shed some light on its function during vertebrate gastrulation. These analyses presented evidence that Cdc42 plays a major role downstream of PTENb signaling pathway in regulating actin dynamics and cellular protrusions in mesoderm [58]. Another study has also showed a link between Cdc42 and p120 Catenin signaling during gastrulation [6]. Moreover, it has been shown that Celf1 (CUGBP Elav-like family member 1), a RNA binding protein, binds several mRNAs including *cdc42*, to control zebrafish somite symmetry, left-right patterning and endoderm organogenesis [59]. Cdc42 is therefore required during early zebrafish morphogenetic events in mesodermal and endodermal cells.

Cdc42 and other Rho GTPases were found to be implicated in germ cell morphology and migration in rodents, mainly in mammalian testis [60]. Moreover, Cdc42 and RhoL are known to control normal transfer of cytoplasm from nurse cells to oocyte in Drosophila [61] and Cdc42 knockdown in C elegans induces disruption of gonad primordium as well as absence of primordial germ cells [62]. As highlighted for Rac1, zebrafish Cdc42 can also impact the behavior of the highly migratory germ cells through its ability to control Blebs’ expansion in migrating germ cells in vivo [63]. This is also relevant for other cell types such as mesendodermal cells [64], however, this is not the case in NC cells, since Syndecan 4 signaling pathway relies on Rac1 activity but not Cdc42 [18].

Paul Martin’s laboratory took advantage of zebrafish larvae transparency and the fact that they present a largely conserved innate immune system in comparison to humans to develop new zebrafish models to image wound-triggered inflammatory response. These studies have revealed important mechanisms by which leukocytes orient towards wound signals in vertebrates. In a zebrafish model of Human Wiskott–Aldrich syndrome (WAS), the migration of macrophages to wounds was significantly reduced [65], however, Cdc42 in this case was not required *per se* for neutrophils migration but was rather essential for WASp regulated phagocytosis [66].

#### 3.1.2. Filopodia and Cilia

One particularity of Cdc42 protein is that, among Rho GTPases proteins, it promotes the formation of cellular protrusions known as filopodia, that are important for cell morphology, cell migration and tissue patterning [67]. A very elegant study led by Eliana Stanganello and her colleagues has shown a crucial role for Cdc42 in actin-based filopodia transport of Wnt8a [68]. In this context, activation of Cdc42 increased the length and frequency of Wnt8a-positive filopodia as well as the expression of many Wnt-targeted genes required for neural plate patterning.

Cdc42 is not only required for filopodia but it can also modify cilia function. Primary cilia are microtubule-based organelles that extend on apical surface of most cells. Their importance is highlighted by a group of diseases that result from defective cilia known as ciliopathies, such as cystic kidney disease, neurodevelopmental abnormalities, blindness, obesity, and perhaps even psychiatric disorders [69]. Zebrafish has also been extensively used for studies of ciliogenesis and kidney development as its kidney pronephros is remarkably simple but functionally conserved [70]. *cdc42* zebrafish morphants present tail curvature, hydrocephalus (abnormal accumulation of fluid in ventricles), eye defects as well as glomerular expansion, that are all features of ciliopathy [71]. Moreover, Cdc42 depletion can lead to a significant increase in retinal cell death and therefore retinal degeneration, that is most likely due to an intracellular transport defect required for ciliogenesis [71,72]. Similar results were obtained in retina-specific Cdc42-knockdown mice, showing that Cdc42 function is well conserved [73]. This phenotype phenocopies *sec10* and *tuba* zebrafish morphants. Indeed, Sec10 is part of the exocyst transport complex that plays an essential role in vesicular transport through its interaction with small GTPases and Tuba is a dynamin binding protein required for ciliogenesis and acts as a GEF for Cdc42 in several processes including ciliogenesis [74].

#### 3.1.3. Angiogenesis

Several studies underscore an essential role for Cdc42 in angiogenesis similar to Rac1 function. Cerebral blood vessels are particularly fragile during development and despite animal models, the biological processes leading to hemorrhage are not well understood. Zebrafish mutants revealed novel pathways required for vascular stability during development. As demonstrated in *redhead* and *bubblehead* mutant, Cdc42 acts upstream of the GEF BPix and its binding partner, Pak2 (P21 (Rac1) Activated Kinase 2), for the maintenance of vascular integrity [75,76]. Wakayama and his colleagues have shown a role, in vivo, for Cdc42 and its partners in angiogenic sprouting of the caudal vein plexus (CVP) in zebrafish [77]. They uncovered a novel signaling pathway that mediates endothelial filopodia extension through an Arhgef9b (Rho guanine nucleotide exchange factor 9)-mediated activation of Cdc42. The latter, in turn, stimulates Fmnl3 (Formin-like protein 3), a member of Formin proteins that promotes filopodia extension and facilitates angiogenesis of the CVP, all of which act downstream of the Bmp signaling. This was quite unexpected since *arhgef9b* deficient mice have normal vascular structures [78]. Similarly, it has been shown that Neuropilin 1 (Nrp1) is required for angiogenic sprouting in the mouse embryonic hindbrain [79], while zebrafish studies revealed novel Cdc42 function during this process. Zebrafish Nrp1 promotes filopodia extension and actin remodeling via Cdc42 activation, more specifically through stimulation with extra cellular matrix (ECM) signaling [80].

### 3.2. Cdc42 in Synapses and Regeneration

One interesting feature of Cdc42 activity is its ability to modulate actin filaments at the synapses. Indeed, mice lacking Cdc42 present defects in postsynaptic plasticity, while its loss of function in presynaptic sensory neurons affects synapse formation [81,82]. Zebrafish was also used as in vivo model to elegantly dissect the role of Cdc42 in actin dynamics at the resolution of a single synapse in a living vertebrate animal [83]. The study investigated the release of the neuropeptide oxytocin from large dense core vesicles in zebrafish and showed a crucial role for Cdc42, downstream of Robo2, in maintaining synaptic oxytocin levels by regulating synaptic actin dynamics. Indeed, Robo2 acts as a GTPase activating protein (GAP) for Cdc42 since it promotes GTP hydrolysis and Cdc42 expression level correlated with oxytocin level in synapses [83].

As discussed for *rac1*, *cdc42* was also identified as a target gene of Wnt5b that is activated during early stages of optic nerve regeneration in zebrafish [34]. A different scenario, however, even though restricted to expression analysis, was observed during zebrafish heart regeneration. In this context, Cdc42 is specifically expressed in ventricular muscle under normal conditions but its expression is unaffected by cardiac injury and regeneration [84], whereas Rac1 expression is not detected under normal conditions but might contribute to cardiomyocyte proliferation during heart regeneration, upstream of Pak2.

## 4. RhoA in Zebrafish Development

Similar to Rac1 and Cdc42, RhoA is one of the most studied small Rho GTPases; it regulates a wide range of cellular processes including cell growth and dynamics [85]. Conditional deletions in mice revealed more specific roles for RhoA in several developmental events such as thymocyte development and erythrocyte survival [86,87]. Being an adequate model to study human physiology and diseases, zebrafish was used to investigate RhoA function in early embryonic development as well as in Ras induced pathologies.

### 4.1. RhoA and Cell Dynamics

Maternal RhoA expression suggested a possible role for this small GTPase in zebrafish early development and morphogenesis. In many animal species, the segregation of the germ plasm (GP), a specialized cytoplasm containing particular mRNAs and proteins [88], is essential for germ cell line determination during early embryonic development. Consistent with its localization during the first two cell division cycles, it has been shown that zebrafish RhoA participates in the establishment of the GP during oogenesis. Pharmacological inhibition of RhoA/ROCK pathway led to microtubules disorganization in zebrafish embryos and to an incorrect localization of the GP during the first divisions [89].

Rho GTPases are main drivers of cell movements in a myriad of morphogenetic events and RhoA is no exception. The first study to establish a direct functional interaction between RhoA and Wnt for cell dynamics regulation in vivo, was assessed in 2006 by Zhu and colleagues [90]. Using morpholino-based specific functional knockdown of RhoA protein, mapping and rescue experiments, the study has unraveled a major positive role of RhoA in CE movements during gastrulation downstream of Wnt11 and Wnt5, similar to its role in xenopus gastrulation [90,91]. Moreover, *rhoa* morphants displayed shortened body axis and were reminiscent of noncanonical Wnt mutants, placing RhoA at the center of the major noncanonical Wnt signaling required for zebrafish CE movements. However, Hsu C. and her colleagues were able to partially rescue gastrulation defects observed in *p120 catenin* morphants by overexpressing a dominant negative form of RhoA, highlighting the necessity of a delicate balance of RhoA activity during gastrulation [6].

As for highly migratory cells, it has been shown that maintaining RhoA activity specifically at the back of NC cells was crucial to locally inhibit Rac activity via Rock. This interaction contributes to the formation of protrusions at the front of NC cells and leads their directional migration [18]. This was the first demonstration of such activity in vivo, thanks to the development of RhoA biosensors and FRET analysis. This is also true for germ cell migration. By applying FRET technique and monitoring actin dynamics, Elena Kardash and her colleagues revealed a role for RhoA in regulating the dynamics of the brushes at the front of germ cells that is required for their migration [19].

Zebrafish embryos undergo the formation of the yolk syncytial layer (YSL) during early development that is vital for the regulation of dorso-anterior axis formation, epiboly and cardiac progenitor cell movements. Overexpression of a constitutive active form of RhoA led to YSL disorganization and mimicked *slc3a2* (solute carrier family 3 member 2) morphants defects, while the attenuation of RhoA activity rescued *slc3a2* morphant phenotype. The authors propose a mechanism in which Slc3a2 inhibits RhoA and contributes to YSL organization via the microtubule network [92]. RhoA was also found to play a role in heart development. *rhoa* mRNA overexpression rescued heart defects observed in *kctd10* (Potassium Channel Tetramerization Domain containing 10) morphants; the latter is a member of the polymerase delta-interacting protein 1 (PDIP1). KCTD10 causes RhoA degradation, a step required for normal cardiogenesis that is likely to be conserved among vertebrates [93].

### 4.2. RhoA and Apoptosis

The suppression of apoptosis by RhoA via diverse anti-apoptotic pathways has been established following several studies. RhoA upregulates anti-apoptotic Bcl2 expression in different cell types such as T cells and osteosarcoma cells [94,95]. This is also the case in zebrafish, Zhu et al. highlighted for the first time, in vivo, an interesting role of RhoA in its ability to prevent apoptosis during embryogenesis via MEK/ERK pathway. They uncovered a genetic link between RhoA, MEK/ERK signaling and Bcl-2 and provided evidence for a role of RhoA in preventing Bcl2-dependent intrinsic apoptosis via activation of MEK/ERK pathway. Zebrafish *rhoa* morphants showed a reduced body size and length and TUNEL assay highlighted a significant increase in apoptosis following gastrulation, a phenotype that was maintained in highly proliferative tissues. The authors propose a link between intensive apoptosis phenotype and reduced body size [96].

### 4.3. RhoA in Pathology and Regeneration

Abnormal RAS signaling is found in up to 30% of all human cancers [97], however adequate animal models that allow a better analysis of RAS signaling crosstalk analyses is still missing. Chew et al. took advantage of zebrafish to generate several transgenic lines in order to study the role of RhoA in RAS- mediated liver tumorigenesis [98]. Liver specific inducible transgenic lines expressing either Kras (Kras^G12V^), a constitutively active form of RhoA (RhoA^G14V^) or a dominant-negative form of RhoA (RhoA^T19N^) were created for this purpose. The study highlighted for the first time a strong crosstalk between Kras and RhoA in liver outgrowth and tumorigenesis via Akt2, p21Cip and S6 kinase. Indeed, while the expression of RhoA^T19N^ expanded liver outgrowth, hepatocellular carcinoma development and cancer mortality, RhoA^G14V^ expression suppressed Kras^G12V^ driven liver overgrowth in zebrafish larvae [98]. This underscores a potential beneficial role for RhoA overexpression in reducing liver tumorigenesis. This was a major breakthrough that highlighted a rather unappreciated role for RhoA in liver tumorigenesis in regards to several previous contradictory in vitro studies [99,100,101,102], underscoring the importance of in vivo models to better understand the role of RhoA signaling in health and disease. Further evidence revealed by Xi Huang and his colleagues linked RhoA activation to the arrest of circulating tumor cells in vivo [103]. Altogether, RhoA stimulation might represent a mechanism for reducing liver tumorigenesis and for halting the circulation of tumor cells in hematogenous metastatic disease.

However, a different outline is proposed during optic nerve regeneration in zebrafish in which RhoA has been reported to be inactivated, contrarily to Rac1 and Cdc42. The authors therefore hypothesize that reducing the levels of RhoA might contribute to optic nerve regeneration [34]. This is also true for spinal cord regeneration in which the microRNA miR-133 plays an important role. The latter interacts with *rhoa* mRNA to reduce its protein levels and contribute to spinal cord regeneration [104]. This is in line with a recent study in Sea Lamprey demonstrating that RhoA knockdown promotes axon regeneration following spinal cord injury [105].

Finally, RhoA activity has been linked to erythroid differentiation since an overexpression of a dominant-negative form of RhoA led to anemia. This phenotype mimicked the knockdown of the RhoA specific guanine nucleotide exchange factor *arhgef12* that could be rescued by a co-injection of a constitutive active form of RhoA (Q63L) [106].

**Figure 2:**
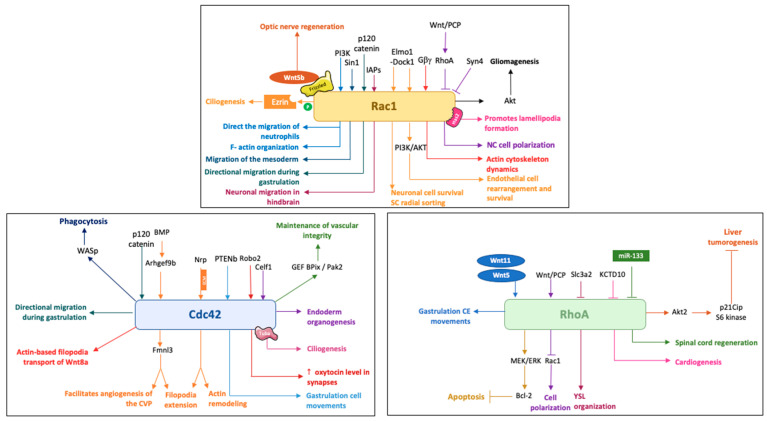
Schematic illustration of the main activities of Rac1, Cdc42 and RhoA in zebrafish and some of their regulators. NC, Neural Crest; SC, Schwann cell.

Studies in zebrafish have shown that the three Rho GTPases control a wide spectrum of developmental functions during which they can play complementary, overlapping or quite distinct functions (Figure 2).

## 5. Zebrafish Tools to Study Small GTPases

It is known from mouse knockout studies that small GTPases are essential for early embryonic morphogenesis since Rac1 and Cdc42 KO mice show early embryonic lethality due to defects in germ layers formation [52,107].

Most studies regarding small GTPases function in zebrafish rely on injecting established constructs that allow either an overexpression of Rac1, Cdc42 and RhoA (via WT or constitutive active (CA) forms) or act as dominant negatives (DN) (see Table 1, Table 2, Table 3, Table 4 and Table 5 below). Some specific morpholinos against *rac1*, *rac1l*, *rac2*, *cdc42* have also been tested and used and the first CRISPR/Cas9 F0 mosaïc cdc42 mutant was created to evaluate its function in the morphogenesis of Müller glia [108], with no obvious phenotype described. Rosowski et al. have generated a loss of function *rac2* zebrafish mutant line and showed a functional redundancy between Rac1 and Rac2 in neutrophils behavior [49].

Conditional or tissue specific manipulation of small GTPases remains the best way to selectively activate or inhibit their function, either by injecting and expressing different constructs for mosaic/single cell analysis or by establishing transgenic lines for a more global analysis in larvae and in adults. Indeed, several transgenic lines that take advantage of the Gal4/UAS system are now available and allow a tissue driven expression of either DN or CA forms of UAS-Rac1, Cdc42 and RhoA when crossed to available Gal4 transgenics under the control of different promoters. Moreover, these transgenics allow you to take full advantage of zebrafish larvae live imaging and circumvent troubles with unavailable antibodies since proteins are tagged with a fluorophore (GFP or mCherry).

Zebrafish is also amenable to pharmacological studies given its external development, therefore several drugs that act as activators or inhibitors of small GTPases have been tested and used in zebrafish larvae. NSC23766 and ML141 remain the most used drugs to inhibit Rac1 activity and Cdc42/Rac1 respectively. Some other compounds have been used as activators of Rac1/Cdc42/RhoA or Rac1 inhibitors but we lack some details as to their specificity and efficacy in zebrafish [46].

Finally, zebrafish is a perfect model to image in vivo protein interactions using fluorescent resonance energy transfer-based probes. Several probes, named Raichu-Rac and Raichu-Cdc42 were designed and used to image these small GTPases activity [109]. Several transgenic lines were generated to study the role of Cdc42 in angiogenesis, some of which contain biosensors: (i) *Tg(UAS:RaichuEV-Cdc42),* FRET-based biosensor, (ii) *Tg(UAS: RaichuEV-Cdc42 NC),* negative control FRET biosensor in which Cdc42 binding domain in PAK1 sequence of RahicuEV-Cdc42 was mutated and (iii) *Tg(UAS:Myr-GFP-ACK42),* Cdc42-specific inhibitor, a cDNA encoding minimal Cdc42 binding domain of human ACK1 (amino acid 504-545, ACK42) [77].

**Table 1:** Available transgenic lines to study Rho GTPases in zebrafish.

Transgenic/Mutant Lines		Observation	Citation
***Rac***			
*Tg(MPO:mCherry-PA-Rac1)^uw^*	Rac1 photoactivation in neutrophils	Rescues protrusion defects in neutrophils following PI(3)K inhibition	[21]
*Tg(UAS:GFP-f2a-Rac1XX)*	UAS driven expression of WT, DN or CA forms of Rac1	XX = WT (^au70^), T17N (^au71^), Q61L (^au72^)	[110]
*Tg(Tol2LUAS-GFP-DARac1-PATol2R)*	UAS driven expression of a Dominant Active form of Rac1	Accelerates gliomagenesis in DAAkt1 fish	[41]
*Tg(mnx1:mcherry-PA-Rac1)*	Rac1 photoactivation in motor neurons	Rescues motor axon guidance defects in *plod3^−/−^*	[33]
*Tg(UAS:EGFP-DN-RAC1)*	UAS driven expression of a DN form of Rac1		[47]
*Tg(UAS:EGFP-CA-RAC1)*	UAS driven expression of a CA form of Rac1		[47]
*Rac2^−/−^*	Loss of function mutation	Motility defects in neutrophils and macrophages	[49]
*Tg(mpeg:mcherry-2A-rac2)*	Rac2 expression in macrophages		[49]
*Tg(mpx:mcherry-2A-rac1)*	Rac1 expression in neutrophils		[49]
*Tg(mpx:mcherry-2A-rac2)*	Rac2 expression in neutrophils		[111]
*Tg(mpx:mcherry-2A-rac2^D57N^)*	Dominant Negative Rac2 mutation in neutrophils	Defective neutrophil wound response	[111]
*Tg(mitfa-V12Rac1GFP)*	Constitutive Active Rac1 expression in melanocytes	Accelerates melanoma progression	[43]
***Cdc42***			
*Tg(UAS: RaichuEV-Cdc42 NC), Negative Control*	UAS driven expression of a Negative Control Cdc42 FRET biosensor		[77]
*Tg(UAS:mCherry-f2a-myc-Cdc42XX)*	UAS driven expression of WT, DN or CA forms of Cdc42	XX = WT (^au66^), T17N (^au67^), Q61L (^au68^), F37A (^au69^)	[110]
*Tg(UAS:RaichuEV-Cdc42)*	UAS driven expression of a Cdc42 FRET-based biosensor		[77]
*Tg(UAS:Myr-GFP-ACK42), Cdc42 inhibitor*	UAS driven expression of a Cdc42 inhibitor FRET biosensor		[77]
***RhoA***			
*Tg(UAS:mCherry-f2a-myc-RhoAXX)*	UAS driven expression of WT, DN or CA forms of RhoA	XX = WT (^au73^), T19N (^au74^), Q63L (^au75^)	[110]
*Tg(fabp10:rtTA2s-M2:TRE2:mCherry-rhoA)*	WT RhoA expression in the liver		[98]
*Tg(fabp10:rtTA2s-M2:TRE2:mCherry-rhoA^T19N^)*	DN RhoA expression in the liver	Accelerates death in *Kras^G12V^* fish with an increase in liver outgrowth and tumorigenesis	[98]
*Tg(fabp10:rtTA2s-M2:TRE2:mCherry-rhoA^G14V^)*	CA RhoA expression in the liver	Negatively regulates Kras oncogenic signaling	[98]

**Table 2:** Available constructs to study Rho GTPases in zebrafish.

Plasmids		Citation
***Rac***		
pCS2-Rac1T17N	Rac1 DN expression	[112]
Tol2-MPO-mCherry-PA-Rac1-polyA	Photoactivated form of Rac1 in neutrophils	[21]
pTol2-Sox10:Rac1V12-P2A-mCherry	CA form of Rac1 in sox10 expressing cells (including Schwann cells)	[37]
mbp:RacV12-P2A-mCherry-CaaX	CA form of Rac1 in mbp expressing cells (Schwann cells and Oligodendrocytes)	[37]
WT-Rac1	WT Rac1 expression	[6]
CA-Rac1	CA Rac1 expression	[4,5,6,8]
Tol2-nxr-UAS-PA Rac1 mcherry	UAS driven expression of a photoactivatable form of Rac1	[31]
DN-Rac1	DN Rac1 expression	[4,5,6,7,9,10,71]
pCS2-rac1l	Rac1l expression	[39]
Rac-FRET-nos1-3′UTR	Rac FRET biosensor	[19,20]
DNRac-nos1-3′UTR	DN form of Rac FRET biosensor	[19,20]
FynTag RFP-T-T2A-Rac1	Rac1 expression	[35]
FynTag RFP-T-T2A-dnRac1	DN Rac1 expression	[35]
pCS2-TagRFP-PBD	Rac1 activity	[10]
psox10: PA-Rac1-mcherry	Photoactivatable form of Rac1 in sox10 expressing cells	[32]
Rac1 (P29S)	Mutant form of Rac1 (Melanoma)	[44]
Rac1- p.C18Y	Mutant form of Rac1 (microcephaly)	[45]
Rac1- p.N39S	Mutant form of Rac1 (microcephaly)	[45]
Raichu-Rac	Rac FRET biosensor	[18,109]
***Cdc42***		
pCS2-cdc42	WT Cdc42 expression	[77]
Cdc42 T17N	DN form of Cdc42	[7,9,71,77]
Cdc42 G12V	CA form of Cdc42	[71,77]
Tol2 UAS:Cdc42-G12V-EGFP	UAS driven expression of a CA form of Cdc42	[83]
Tol2 UAS:Cdc42- T17N-EGFP	UAS driven expression of a DN form of Cdc42	[83]
Raichu-Cdc42	Cdc42 FRET biosensor	[18,109]
***RhoA***		
RhoA biosensor	RhoA FRET biosensor	[18,113]
RhoAT19N	DN form of RhoA	[98]
RhoAV14	CA form of RhoA	[6,98]
WT-RhoA mRNA	WT RhoA expression	[6]
RhoA Q63L mRNA	CA form of RhoA	[106]

**Table 3:** Available morpholinos (MOs) and tools to activate or inhibit Rho GTPases activity in zebrafish.

Morpholinos/gRNA/Drug Inhibitors or Activators	Sequence	Citation
***Rac***		
siRac1ss,	CCCUAACACUCCAAUAAUUtt	[34]
Rac1as,	AAUUAUUGGAGUGUUAGGGtt	[34]
siRNAcontrol1ss,	UAGCCCACACCACGAUAGAtt	[34]
siRNAcontrol1as,	UCUAUCGUGUGGGCUAtt	[34]
siRNAcontrol2ss,	UCGACCAGGGCGGAUGUGtt	[34]
siRNAcontrol2as,	CACUAUCCGCCCUGGUCGAtt	[34]
NCS23766 (Rac1 inhibitor)		[23,44,48]
Rac1 inhibitor I		[48]
Rac1 inhibitor II		[48]
TB-MO rac1l	5′-CCACACACTTGATGGCCTGCATGAC-3′	[39]
TB-MO rac1	5′-CCACACACTTTATGGCCTGCATCTG-3′	[39]
TB-MO rac1	5′-GCCTGCATGGCAGCGAATGTCCCG-3′	[39,91]
Activator of Rho/Rac/Cdc42 I		[46]
Activator of Rac/Cdc42 II		[46]
***Cdc4*** *2*		
Cdc42 gRNA#2	GGGAGACGACTTCTTAACAGTGG	[108]
ML141 (Cdc42/Rac1 inhibitor)		[80]
ACK42	Cdc42-specific inhibitor, a cDNA encoding minimal Cdc42 binding domain of human ACK1 (amino acid 504–545)	[77]
siCDC42ss,	GGGUAAAACCUGUCUAUUAtt	[34]
siCDC42as,	UAAUAGACAGGUUUUACCCtt	[34]
***RhoA***		
RhoA MO1	5′-TCCGTCGCCTCTCTTATGTCCGATA-3′	[90]
RhoA MO2	5′-CTAGCCGTTTTGTTTTAGTCCAACG-3′	[90]

**Table 4:** Available antibodies for immunostaining of Rho GTPases in zebrafish.

Antibodies	Reference	Citation
***Rac***		
Anti Rac1	Upstate biotechnology	[38]
Anti-Rac1	Millipore 05-389	[114]
Anti-Rac1	Abcam	[84]
Anti-Rac1	Santa Cruz Biotechnology (C14, sc-217)	[89]
***Cdc42***		
Mouse monoclonal anti-Cdc42 (610929)	BD Transduction Laboratories)	[71]
Anti-Cdc42	(Lifespan Biologicals)	[84]
Anti-Cdc42	BD	[77]
***RhoA***		
Anti-RhoA	Santa Cruz Biotechnology (119, sc-179)	[89]
Anti-RhoA-GTP		[89]

**Table 5:** Designed primers to study Rho GTPases expression in zebrafish.

Primers for qPCR	Sequence	Citation
***Rac***		
Rac1-F	GGTGAATCTGGGCTTATGGG	[47]
Rac1-R	TCAGGATACCACTTTGCACG	[47]
Rac2-F	ACTCTCCTACCC GCAGACG	[111]
Rac2-R	CACCTCTGGGTACCACTTGGC	[111]
***Cdc42***		
Zeb cdc42-F	5′-GACAGTAGCCCTGTAAATGGTTG-3′	[59]
Zeb cdc42-R	5′-GTTAGAAAGTTCCCTGCTTGAGAG-3′	[59]
***RhoA***		
qrhoaa_F	TCCTGAGGTTTACGTTCCCA	[49]
qrhoaa_R	TGGCCAGCTGTATCCCATAG	[49]
qrhoab_F	ACAGGCTTCGTCCTCTTTCA	[49]
qrhoab_R	AATGTTTGACCTCAGGCGTC	[49]

## 6. Conclusions

Zebrafish is a remarkable vertebrate model for in vivo live imaging that helped us unfold several aspects of Rho GTPases function over the past decade. Work performed so far has taught us a great deal about how these small GTPases regulate dynamic changes within numerous cell types and at different developmental stages. The ability to follow Rho GTPase signaling and to locally modulate its function in real time, combined with the rapidly evolving imaging and genetic techniques, will ensure that the zebrafish remains a key genetic model for study of these small proteins in cell dynamics and signaling in vivo. Generating more zebrafish tools will help us understand not only the role of Rho, Rac and Cdc42 in development and disease but also how their regulators as well as other atypical Rho family members, that have been neglected so far, contribute to these processes.
